# Effectiveness of mouthwashes to reduce the SARS-COV-2 load in saliva of adults with diagnosis of COVID-19: Systematic review and meta-analysis

**DOI:** 10.4317/jced.62196

**Published:** 2025-01-01

**Authors:** Rosita Elena Espejo-Carrera, Angel Steven Asmat-Abanto, Marcos Jimmy Carruitero-Honores, José Antonio Caballero-Alvarado

**Affiliations:** 1 Master’s degree in Clinical Research Sciences. Professor of the Posgraduate School, Universidad Privada Antenor Orrego, Trujillo, La Libertad, Peru. Professor of Stomatology Study Program, Universidad Privada Antenor Orrego, Trujillo, La Libertad, Peru; 2 Doctor in Stomatology, Specialist in Periodontics. Professor of Human Medicine Study Program, Universidad Privada Antenor Orrego, Trujillo, La Libertad, Peru. Professor of Stomatology Study Program, Universidad Privada Antenor Orrego, Trujillo, La Libertad, Peru; 3 Postdoctoral Fellow, Specialist in Orthodontics. Professor of Human Medicine Study Program, Universidad Privada Antenor Orrego, Trujillo, La Libertad, Peru; 4 Doctor in Clinical and Translational Research, Specialist in General Surgery. Professor of Human Medicine Study Program, Universidad Privada Antenor Orrego, Trujillo, La Libertad, Peru. Professor of Posgraduate School, Universidad Privada Antenor Orrego, Trujillo, La Libertad, Peru. Physician of Surgery Department, Regional Hospital of Trujillo, Trujillo, La Libertad, Peru

## Abstract

**Background:**

COVID-19 still represents a threat to public health. In this sense, antiseptic mouthwashes have been suggested to reduce cross-contamination and community transmission.

**Material and Methods:**

This systematic review and meta-analysis aimed to synthesize the evidence on the effectiveness of povidone-iodine (PVP-I), cetylpyridinium chloride (CPC) and chlorhexidine (CHX) mouthwashes in reducing SARS-COV-2 viral load in the saliva of adults diagnosed with COVID-19. After the systematic search in five electronic databases, 16 clinical trials published until June 2023 were analyzed. Of these, 6 were included in the meta-analysis.

**Results:**

The standardized mean difference (SMD) was reported with its corresponding 95% confidence intervals (95%CI). An overall SMD of 0.51 (95%CI: -0.29 to 1.32; I² = 46.0%; *p* = 0.047) was found. For CPC, the combined effect found in the studies was not significant (SMD = -0.07; 95%CI: -0.42 to 0.28; I² = 0.0%; *p* = 0.373); the same occurred for CHX (SMD = 0.50; 95%CI: -43.32 to 44.32; I² = 0.0%; *p* = 1.000). However, PVP-I showed a more consistent profile with a significant combined effect (SMD = 4.15; 95%CI: 2.11 to 6.18) and negligible heterogeneity (I² = 0.0%; *p* = 0.908).

**Conclusions:**

The findings indicate a non-significant effect of mouthwashes on reducing viral load when all types were evaluated together. Separately, only PVP-I showed a significant reduction in viral load with a low level of certainty of evidence, while for CPC and CHX the reduction was not significant, with a low and very low level of certainty of evidence, respectively.

** Key words:**SARS-CoV-2, mouthwashes, povidone-iodine, cetylpyridinium, chlorhexidine.

## Introduction

COVID-19 has had a great impact on public health due to its rapid spread and the lack of effective measures to prevent infections or reduce their severity (1). It is caused by the Severe Acute Respiratory Syndrome Coronavirus type 2 (SARS-CoV-2) and is transmitted by the respiratory route through microdroplets or by direct contact with contaminated surfaces (2,3), causing atypical pneumonia with possible involvement of multiple organs and body systems (4).

The main source of SARS-CoV-2 transmission is symptomatic patients, whose viral load in saliva is highest in the first week after the onset of symptoms. However, asymptomatic and presymptomatic patients also have the capacity to be contagious (5,6). Likewise, the viral load in saliva is associated with the severity of COVID-19 and is considered a predictor of death, and it is of even greater importance than the patient’s age (7). This is because the main cellular receptor of the virus is angiotensin-converting enzyme 2 (ACE2), which has a high level of expression in the oral mucosa, particularly in the epithelium of the tongue and salivary glands (8-11). In this sense, the viral load attains a number of up to 1.2x 108 copies/mL and is present in 91.7% of saliva samples from individuals diagnosed with COVID-19 (12,13).

As saliva is the main infection route, oral antiseptics could be very useful to reduce the burden of SARS-CoV-2 and reduce transmission between individuals (2,4,9,11). Moreover, there have been reports indicating that by reducing the viral load in saliva by means of using mouthwashes containing CHX, CPC, and PVP-I, the severity of the disease would be reduced in terms of hospitalization time, admission to intensive care, and death. (2,3,7,8). This could be useful for dentistry and medical specialties, in which procedures involve generating aerosols and working close to the patient. These professionals would be directly and constantly exposed to this infection (14,15), implying a risk to their health and community.

Because individual studies may not have sufficient statistical power to reach a reliable conclusion, and the majority of systematic reviews found included studies with heterogeneous designs; the present systematic review and meta-analysis was conducted as an update on the topic, with the aim of synthesizing the evidence on the effectiveness of CHX, CPC and PVP-I-based mouthwashes to reduce the load of SARS-CoV-2 in saliva of adult patients with a diagnosis of COVID-19.

## Material and Methods

-Protocol and Registration

The present systematic review was registered at the Postgraduate School of the Universidad Privada Antenor Orrego and approved by the Permanent Research Committee (Resolution No. 0800-2022-D-EPG-UPAO). It was conducted in accordance with the Preferred Reporting Items for Systematic Reviews and Meta-analyses checklist (PRISMA, 2020) (16).

-Focused Question

The research question was as follows: Do povidone-iodine, cetylpyridinium chloride, or chlorhexidine mouthwashes reduce the load of SARS-CoV-2 in the saliva of adults diagnosed with COVID-19? This was proposed according to the PICOD strategy for research (population/patients, intervention, comparison, results, and design). Where *P* = adult patients diagnosed with COVID-19, I = povidone-iodine, cetylpyridinium chloride or chlorhexidine mouthwashes, C = placebo or no intervention, R = SARS-CoV-2 load reduction in saliva, and D = randomized controlled clinical trials

-Eligibility criteria and process of selection 

Randomized, controlled, parallel-arm clinical trials were included, using distilled/sterile/tap water, saline, or no treatment as a control group, which specified dosage, duration, or frequency of mouthwashes and who measured loads as in saliva in terms of copies/ml or cycle threshold (Ct) values, before and after the intervention using PCR. Studies in which another treatment was added to the use of mouthwash or had incomplete data were excluded.

-Search Strategy

The search was conducted in the PubMed/Medline, Web of Science, Scopus, Embase, and BVS databases in December 2022 and updated in June 2023, in addition to manual searches in the reference lists of all studies included and previously published reviews. The following search terms were used: (COVID-19 OR coronavirus OR SARS-CoV-2) AND (mouthwash* OR povidone-iodine OR PVP-I OR cetylpyridinium OR chlorhexidine) AND (“viral load” OR “viral burden” OR “virus titer”). This was adapted according to the syntax rules of each database ( Table 1).

Data extraction, risk of bias, and certainty of evidence

The literature search results were uploaded to the Rayyan Systematic Reviews Application (17) and duplicate records were removed. Two researchers (R.E.C. and A.A.A.) independently selected the articles to be analyzed, first by title and abstract, then by full text. Any disagreement was discussed with the participation of a third researcher (M.C.H.). Data were then extracted independently into an Excel spreadsheet (Microsoft® Excel® for Office 365). Subsequently, the articles selected and the data extracted were reviewed and approved by a fourth expert researcher (J.C.A.). Moreover, the clinical trials included were analyzed using the Cochrane Collaboration’s RoB 2.0 tool to assess the risk of bias (18).

Disagreements were resolved with the collaboration of the third and fourth investigators. The quality of evidence of the studies included in the meta-analysis was assessed using the GRADE tool, using the GRADEpro GDT software (19).

-Summary of Results

All outcome measures that assessed the reduction of SARS-CoV-2 viral load in saliva were considered. The information required for each study was collected in preliminary summary Tables. If these data were not found in the article, an email was sent to the authors to request them. Results with sufficient data to calculate an estimate of the effect were used for meta-analysis.

## Results

-Selection of Studies 

**Figure 1:**
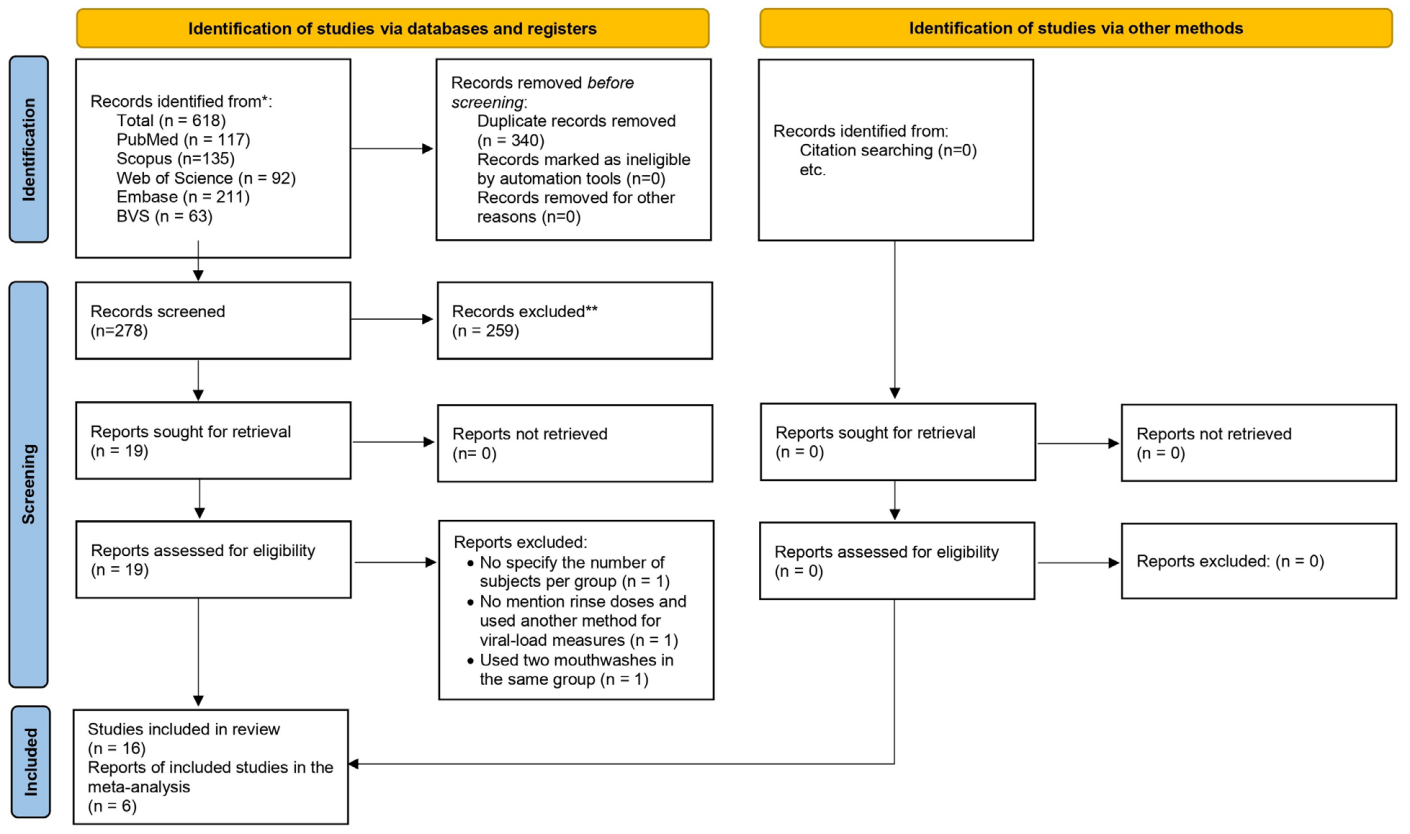
PRISMA 2020 flow diagram showing the entire search process.

As presented in the PRISMA 2020 flowchart (20) (Fig. 1), 618 records were retrieved. After the removal of duplicates and selection by title and abstract, 19 articles remained for full-text evaluation. Of these, 3 were excluded for the following reasons: they did not specify the number of patients evaluated per study arm, they did not mention the dose of a rinse used and they used a method other than PCR to measure viral load and they used two of the mouthwashes in the same study arm. Finally, the qualitative analysis was carried out with 16 studies ( Table 2), 6 of which were chosen for the meta-analysis.

-Characteristics of the Studies

In the 16 studies, a total of 919 subjects were evaluated. Those with the smallest number of participants evaluated 16 patients (3,21), and the study with the highest number evaluated 120 (10). The concentrations used for PVP-I were 0.25%, 0.5%, 1%, and 2%; for CPC, they were 0.07% and 0.075%. and for CHX, 0.12% and 0.2%. Three studies (3,5,22) compared PVP-I, CPC, and CHX; seven (8,21,23-27) compared at least two of them; and six (10,28-32) only one of them with the use of other study groups or placebo. All of them measured the viral load in saliva before and after the intervention at variable time intervals, the minimum time being immediately after rinsing and the maximum time interval after 6 hours.

The studies analyzed were conducted in Spain (5,22,28,31,32), Brazil (24,29), United States (21,23), Saudi Arabia (26,27), Italy (25), Singapore (3), Turkey (30), Lebanon (8) and Iran (10). The ages of the subjects studied ranged between 20 and 83 years old. Seven of the studies (3,5,10,22,25,26,32) did not mention adverse drug reactions. With respect to possible conflicts of interest, two (28,31) declared financing by DENTAID SL., one (24) by Colgate-Palmolive Company and another (23) did not submit the declaration. Seven of the studies (5,8,10,22,24,25,30) were conducted with hospitalized patients, and the other nine (3,21,23,26-29,31,32) were conducted with outpatients.

-Meta-analysis of the synthesis

**Figure 2:**
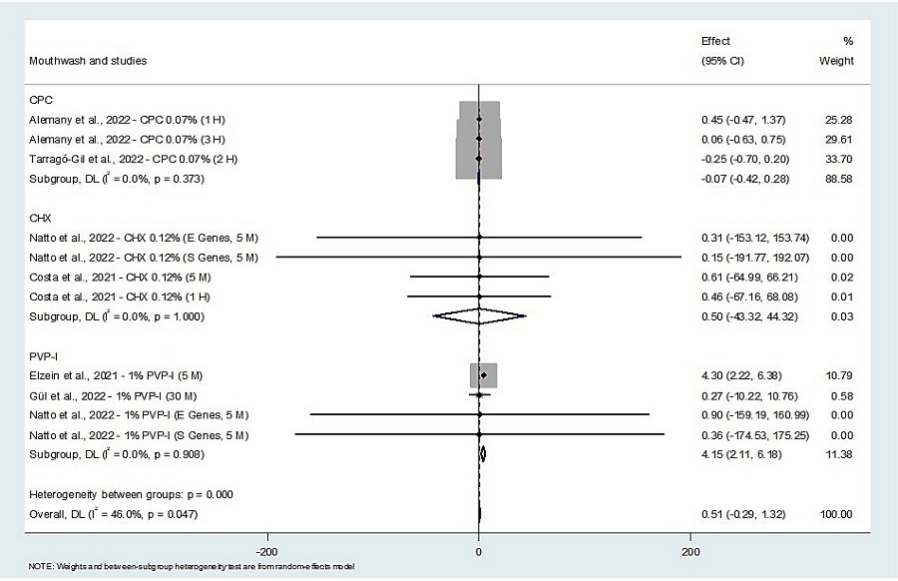
Forest plot of mouthwashes effects in reducing the viral load of SARS-CoV-2 in saliva. (Legend: *CPC = cetylpyridinium chloride, CHX = chlorhexidine, PVP-I = povidone-iodine.).

The results of the meta-analysis are presented in the forest plot of Fig. 2, in which the standardized mean difference (SMD) is observed as a measure of the effect. Overall, a mean of 0.51 SMD (95% CI: -0.29; 1.32), which reflects the standardized effect size for all studies (I² = 46.0%; *p* = 0.047).

The studies included that evaluated CPC and CHX offered mixed results. For CPC, Alemany *et al*. (28) found an effect of 0.45 when they measured at one hour and 0.06 at three hours, while Tarragó-Gil *et al*. (31) reported an effect of -0.25 for a measurement after two hours. The subgroup analysis for CPC showed a non-significant medium combined effect (-0.07); likewise, the variability of the studies was not significant (I² = 0.0%; *p* = 0.373) with low certainty of evidence. With respect to CHX, Natto *et al*. (26) observed effects of 0.31 and 0.15 for specific genetic variants, while Costa *et al*. (29) reported effects of 0.61 and 0.46 for different measurement time intervals. The CHX subgroup had a non-significant mean combined effect (0.50), with non-significant perfect homogeneity (I² = 0.0%; *p* = 1.000) and very low certainty of evidence.

For the PVP-I-based mouthwashes, the results were more homogeneous. Elzein *et al*. (8) showed an effect of 4.30, while Gül *et al*. (30) reported a more modest effect (0.27). The studies of Natto *et al*. (26) also supported the effectiveness of PVP-I with effect sizes of 0.90 and 0.36. The subgroup analysis for PVP-I concluded with a significant mean combined effect in viral load reduction (4.15), with very low heterogeneity between studies, statistically non-significant (I² = 0.0%; *p* = 0.908), and low certainty of evidence.

Egger’s regression indicated the absence of publication bias (*p*=0.56). It is worth mentioning that the meta-analysis initially included seven studies. However, after performing the sensitivity analysis to evaluate the robustness of the results, the researchers found that the study by Fantozzi *et al*. (25) reported their values in medians and interquartile ranges, unlike the rest of the studies selected, which reported them in means and standard deviations. Since it significantly altered the overall results, it was excluded from the main combined analysis. This exclusion led to greater consistency in the results. Despite this exclusion, the variability of reporting methods was considered an important factor in the qualitative interpretation of the results.

Risk of bias and quality of evidence

**Figure 3:**
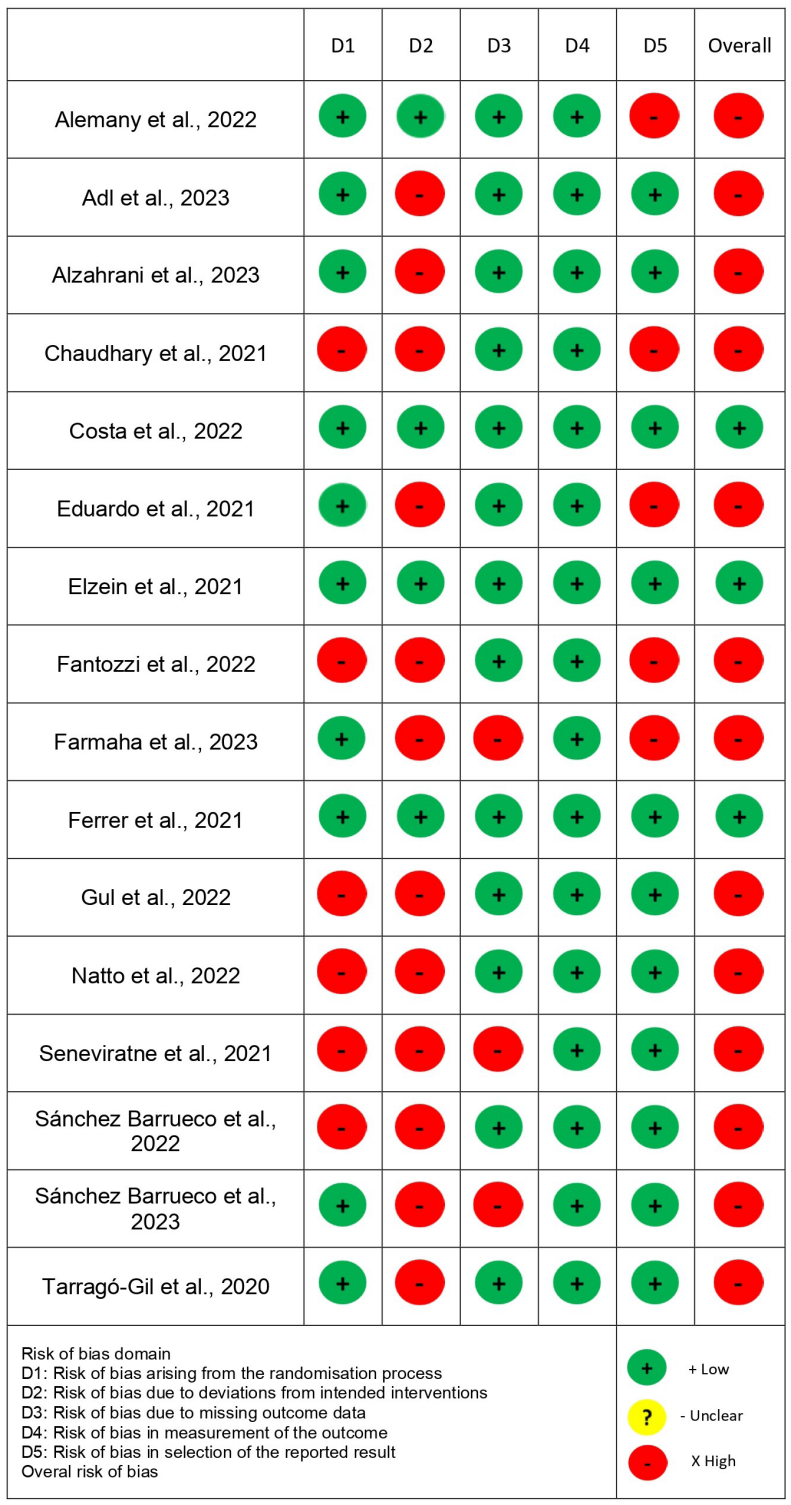
Summary of the risk of bias assessment – Cochrane tool (RoB 2.0).

The RoB 2.0 tool was used for risk of bias assessment (Fig. 3). Three studies presented low risk (5,8,29), and the others presented high risk of bias. The quality of the evidence of the studies included in the meta-analysis, according to GRADE, is presented in  Table 3.

## Discussion

COVID-19 continues to be a threat that requires the development of effective and cost-effective intervention measures to prevent cross-contamination and community transmission (33). This systematic review with meta-analysis aimed to evaluate the effectiveness of three mouthwashes in reducing the viral load of SARS-CoV2 in the saliva of adult patients diagnosed with COVID-19.

In the clinical trials analyzed, CHX was the substance most frequently studied, followed by PVP-I and CPC. Ferrer *et al*. (5), Sánchez *et al*. (22), and Seneviratne *et al*. (3) included all three mouthwashes in their studies. The first two found no differences when compared with the controls; while Seneviratne *et al* (3) observed a significant decrease in viral load between groups, at 5 minutes for CPC and at 6 hours for CPC and PVP-I, compared with the control. It is worth mentioning that they only worked with 2 subjects as a control group. The three studies had a small sample size and no statistical basis. Attention is drawn to the fact that in the two studies in which the mouthwashes did not affect the viral load (5,22), the concentration of PVP-I was much higher (PVP-I 2%) than in the study where an effect was obtained (PVP -I 0.5%) (3). In the latter, the rinsing time was even shorter. Only the study of Ferrer *et al*. (5) showed a low risk of bias, while the other two studies had a high risk of bias. Therefore, it could not yet be concluded that there was real effectiveness; in agreement with the systematic review by Hernández-Vásquez *et al*. (1) but disagreeing with the reviews by Mezarina *et al*. (2), García-Sánchez *et al*. (4) and Ziaeefar *et al*. (34), possibly because they evaluated a smaller number of studies and with methodological deficiencies as they were the first clinical trials. In contrast, Ting *et al*. (35), despite declaring the effectiveness of these mouthwashes, mentioned the importance of distinguishing whether they were better than water and saline solution. These statements were contradictory.

Farmaha *et al*. (21), Elzein *et al*. (8), Chaudhary *et al*. (23), Natto *et al*. (26), and Fantozzi *et al*. (25), only compared two of the mouthwashes of our interest, CHX and PVP-I. Farmaha *et al*. (21) and Elzein *et al*. (8) found the effectiveness of both mouthwashes compared with the control immediately after and after 5 minutes, respectively. Furthermore, for Farmaha *et al*. (21), CHX maintained its effect for up to 2 hours. However, Chaudhary *et al*. (23), Natto *et al*. (26) and Fantozzi *et al*. (25), concluded that none of the mouthwashes demonstrated effectiveness. All the studies mentioned had a very limited number of subjects per study group, which would affect the internal validity of the clinical trials. Moreover, their results have been reported confusingly, with the exception of the study of Elzein *et al*. (8), which showed a low risk of bias. These findings were consistent with the systematic reviews and meta-analyses of Zhang *et al*. (36) and Hasan *et al*. (37), who concluded that both PVP-I and CHX were effective, mainly within the first 30 minutes after rinsing, and that PVP-I was better.

Eduardo *et al*. (24) evaluated CPC and CHX and reported that both reduced the viral load immediately afterward, at 30 min, and up to 1 hour later. However, it is important to assess the conflict of interest reported by the authors. In contrast, Ebrahimi *et al*. (38), in their systematic review and meta-analysis, indicated that there was insufficient evidence about the effects of CPC and CHX on the reduction of SARS-CoV-2 viral load. Alzahrani *et al*. (27) evaluated PVP-I and CPC and found differences between the groups and the control with distilled water versus the control without rinsing at 60 minutes, which would imply a mechanical washing effect but not an antiviral effect. The aforementioned studies showed a high risk of bias.

Among the studies that evaluated at least one of the mouthwashes required for this review, Tarragó-Gil *et al*. (31) and Alemany *et al*. (28) found no differences between the CPC and control groups. It is important to emphasize that the sample size for these studies was larger than forty patients per group, which would mean an improvement in statistical power compared with most studies analyzed. In this regard, Sbricoli *et al*. (39), in their systematic review and meta-analysis, observed inconsistent results regarding the reduction of viral load in saliva induced by this active ingredient, while D’Amico *et al*. (7), in their systematic review, indicated that CPC was effective.

Costa *et al*. (29) and Sánchez *et al*. (32) reported that CHX was effective in decreasing the viral load in saliva for at least 1 hour. Moreover, Sánchez *et al*. (32), when comparing two concentrations of CHX, 0.12% and 0.2%, unexpectedly found the lower concentration to be more effective, indicating that the ingredients that accompany the active principle could have some effect on the results. These two studies showed a small number of participants, and only that of Costa *et al*. (29) showed a low risk of bias. Therefore, studies with better methodological design are required. These findings contradict the systematic review by Sbricoli *et al*. (39), who indicated that CHX at 0.2% was associated with a reduction in viral load, agreeing with Fernández *et al*. (40) and Rahman *et al*. (14), who also mentioned that the effect lasted for a short period, and Ting *et al*. (35), who reported a maximum reduction after 60 minutes.

The studies of Gül *et al*. (30) and Adl *et al*. (10), found that PVP-I was not effective in reducing the viral load. The risk of bias for both was high; for this reason, it is also recommended that studies with better methodology and larger sample sizes be conducted. Conversely, Ebrahimi *et al*. (38), in their systematic review, recommended the use of PVP-I mouthwashes to reduce the viral load of SARS-COV-2 in the oral cavity of patients before and during dental procedures.

Some of the clinical trials analyzed in this systematic review’s results were contradictory. Chaudhary *et al*. (23), Fantozzi *et al*. (25), and Ferrer *et al*. (5) observed significant reductions in viral loads in their control groups; while in the study by Alzaharani *et al*. (27), a reduction was observed in the control group compared with the group without rinsing. These results suggested a possible mechanical washing effect of viral particles due to the rinsing process.

The meta-analysis showed a significant combined effect only for PVP-I, (8,26,30) while for CPC (28,31) and CHX (26,29), the effects were not significant. However, taking into consideration the level of certainty of the evidence according to GRADE, these results would indicate that confidence in the effect estimate was limited or unreliable; that is, the true effect could differ substantially from the estimated effect. Moreover, it is important to mention the variability in the allocation times of the mouthwashes; for PVP-I, the measurement was immediately and 5 minutes after the rinse, and for CPC and CHX, the measurements were recorded between 1 and 3 hours after the rinse.

Within the limitations of this review, it should be considered that the majority of clinical trials conducted to date showed inadequate sample sizes, high risk of bias, variable intervention times, and even some studies with a possible conflict of interest, making it difficult to perform a more robust meta-analysis. However, randomization for the distribution of treatments and control groups could be considered a strength of the analyzed studies. In general, the recommendation to conduct clinical trials with a better methodological design and a larger number of patients is reiterated. As an additional point, the studies included only evaluated the presence of viral particles but not their viability or capacity for being infectious.

The present systematic review and meta-analysis concluded that when evaluating all mouthwashes simultaneously, no significant effect was found. However, when evaluated separately, only PVP-I effectively reduced the SARS-CoV-2 load in saliva. The results should be considered with great caution due to the high risk of bias shown in the clinical trials analyzed and the low level of certainty of the evidence.

## Figures and Tables

**Table 1:** Search strategy in the databases.

Database	Search strategy
PubMed	1,((COVID-19) OR (coronavirus)) OR (SARS-CoV-2),,,"""covid 19""[All Fields] OR ""covid 19""[MeSH Terms] OR ""covid 19 vaccines""[All Fields] OR ""covid 19 vaccines""[MeSH Terms] OR ""covid 19 serotherapy""[All Fields] OR ""covid 19 nucleic acid testing""[All Fields] OR ""covid 19 nucleic acid testing""[MeSH Terms] OR ""covid 19 serological testing""[All Fields] OR ""covid 19 serological testing""[MeSH Terms] OR ""covid 19 testing""[All Fields] OR ""covid 19 testing""[MeSH Terms] OR ""sars cov 2""[All Fields] OR ""sars cov 2""[MeSH Terms] OR ""severe acute respiratory syndrome coronavirus 2""[All Fields] OR ""ncov""[All Fields] OR ""2019 ncov""[All Fields] OR ((""coronavirus""[MeSH Terms] OR ""coronavirus""[All Fields] OR ""cov""[All Fields]) OR (""coronavirus""[MeSH Terms] OR ""coronavirus""[All Fields] OR ""coronaviruses""[All Fields]) OR (""sars cov 2""[MeSH Terms] OR ""sars cov 2""[All Fields] OR ""sars cov 2""[All Fields])" 2,(((mouthwashes) OR (povidone-iodine)) OR (cetylpyridinium)) OR (chlorhexidine),,,"""mouthwashes""[Pharmacological Action] OR ""mouthwashes""[MeSH Terms] OR ""mouthwashes""[All Fields] OR ""mouthwash""[All Fields] OR ""mouthwashing""[All Fields] OR ""mouthwashings""[All Fields] OR (""povidone iodine""[MeSH Terms] OR ""povidone iodine""[All Fields] OR (""povidone""[All Fields] AND ""iodine""[All Fields]) OR ""povidone iodine""[All Fields]) OR (""cetylpyridinium""[MeSH Terms] OR ""cetylpyridinium""[All Fields]) OR (""chlorhexidine""[MeSH Terms] OR ""chlorhexidine""[All Fields] OR ""chlorhexidin""[All Fields])" 3,viral load,,,"""viral load""[MeSH Terms] OR (""viral""[All Fields] AND ""load""[All Fields]) OR ""viral load""[All Fields]" 4,#1 AND #2 AND #3
Scopus	TITLE-ABS-KEY((COVID-19 OR coronavirus OR SARS-CoV-2) AND (mouthwash* OR povidone-iodine OR PVP-I* OR cetylpyridinium OR chlorhexidine) AND ("viral load" OR "viral burden" OR "virus titer"))
Web of Science	1: ALL=(COVID-19 OR coronavirus OR SARS-CoV-2) 2: ALL=(mouthwash* OR povidone-iodine OR PVP-I OR cetylpyridinium OR chlorhexidine) 3: ALL=("viral load" OR "viral burden" OR "virus titer") 4: #1 AND #2 AND #3
Embase	("COVID-19 OR coronavirus OR SARS-CoV-2) AND (mouthwash* OR povidone-iodine OR PVP-I* OR cetylpyridinium OR chlorhexidine) AND ("viral load" OR "viral burden" OR "virus titer")
BVS	(COVID-19 OR coronavirus OR SARS-CoV-2) AND (mouthwash* OR povidone-iodine OR PVP-I OR cetylpyridinium OR chlorhexidine) AND ("viral load" OR "viral burden" OR "virus titer")

**Table 2:** General characteristics of included studies.

Authors (year)	Country	Study Design	Number of participants evaluated	Number of patients per study arm	Saliva sample collection	Intervention Group (Type of mouthwash, dosage, and duration of rinse)	Control Group	Viral load test	Results
Adl et al. (2023)	Iran	Pilot, randomized, double-blind, multicenter, parallel, controlled	120	Group 1: hospitalized Subgroup HP (n=20) Subgroup PVP-I (n=20) Subgroup control (n=20) Group 2: outpatients Subgroup HP (n=20) Subgroup PVP-I (n=20) Subgroup control (n=20)	At the beginning and 10 minutes after gargling.	HP 1%, 10 ml for 30 s. PVP-I 0.25%, 10 ml for 30 s.	Saline solution, 10 ml for 30 s.	RT-qPCR	HP or PVP-I mouthwashes did not reduce SARS-CoV-2 viral load in saliva.
Alemany et al. (2022)	Spain	Randomized, double-blind, parallel, controlled, multicenter	105	CPC Group (n=51) Control Group (n=54)	At the beginning of the study, 1 and 3 hours after rinsing.	CPC 0.07% 15 ml for 1 min.	Distilled water 15 ml for 1 min.	RT-PCR ELISA quantitative	They found no significant differences between the groups at 1 hour or 3 hours.
Alzahrani et al. (2023)	Saudi Arabia	Randomized, double blind, parallel, controlled	55	Grupo PVP-I (n=6) Grupo HP ((n=11) Grupo CPC (n=11) Grupo HOCl (n=9) Grupo control 1 (n=8) Grupo control 2 (n=10)	At the beginning, 5, 30 minutes, and 1 hour after rinsing.	PVP-I 1%, 15 ml for 30 s. HP 1.5%, 15 ml for 30 s. CPC 0,075%, 15 ml for 30 s. HOCl 80ppm, 15 ml for 30 s.	Control 1: Distilled water, 15 ml for 30 s. Control 2: without rinsing	RT-qPCR	Only HP showed a significant reduction at all three times. PVP-I, HP, CPC, and the control group with distilled water showed an effect within one hour, compared with the group without rinsing.
Chaudhary et al. (2021)	United States of America	Randomized, triple-blind, parallel, controlled	40	Group HP (n=10) Group CHX (n=10) Group PVP-I (n=10) Control Group (n=10)	At the beginning of the study, 15 and 45 minutes after rinsing.	HP 1%, 15 ml for 1 min. CHX 0.12%, 15 ml for 1 min. PVP-I 0.5%, 15 ml for 1 min.	Saline solution 15 ml for 1 min.	RT-qPCR	Mean viral load was reduced from 61% to 89% at 15 minutes and from 70% to 97% at 45 minutes for all mouthwashes.
Costa et al. (2022)	Brazil	Randomized, double-blind, parallel, controlled	100	Group CHX (n= 50) Control Group (n=50)	At the beginning of the study, 5 minutes and 1 hour after rinsing	CHX 0.12%, 15 ml for 1 min.	Placebo (not specified) 15 ml for 1 min.	RT-qPCR	CHX was effective in decreasing the SARS-CoV-2 viral load in saliva for at least 1 hour.
Eduardo et al. (2021)	Brazil	Pilot, randomized, double-blind, parallel, controlled	43	Group CPC (n=7) Group HP (n=7) Group CHX (n=8) Group HP + CHX (n=12) Control Group (n=9)	At the beginning of the study, immediately after, at 30 minutes and 1 hour after rinsing.	CPC 0.075% 20 ml for 30 s. HP 1.5%, 10 ml for 1 min. CHX 0.12%,15 ml for 30s HP 1.5%, 10 ml for 1 min, followed by CHX 0.12%, 15 ml for 30 s.	Distilled water 20 ml for 1 min.	RT-qPCR	CPC and CHX significantly reduced viral load up to 1 hour after rinsing, while HP significantly reduced up to 30 minutes after rinsing.
Elzein et al. (2021)	Lebanon	Randomized, triple-blind, parallel, controlled	61	Group CHX (n=27) Group PVP-I (n=25) Control Group (n=9)	At the beginning of the study and 5 minutes after rinsing.	CHX 0.2%,15 ml for 30s PVP-I 1%, 15 ml for 30 s.	Distilled water 15 ml for 30 s.	rRT-PCR	Both solutions demonstrated significant efficacy against SARS-CoV-2 in saliva. Distilled water did not affect viral load.
Fantozzi et al. (2022)	Italy	Pilot, randomized, single-blind, controlled	38	Group CHX (n=8) Group PVP-I (n=8) Group HP (n=11) Control Group (n=11)	At the beginning of the study, immediately after rinsing and after 45 minutes.	CHX 0.12%, 15 ml for 1 min. PVP-I 1%, 15 ml for 1 min. HP 1%, 15 ml for 1 min.	Sodium chloride (0.9%), 15 ml for 1 min.	rRT-PCR	None of the rinses showed statistically significant reductions.
Farmaha et al. (2023)	United States of America	Randomized, double-blind, parallel, controlled	16	Group CHX (n=3) Group HP (n=4) Group PVP-I (n=3) Group Listerine (n=3) Control Group (n=3)	At the beginning and immediately after, 1 and 2 hours after rinsing.	CHX 0.12%, 5 ml for 2 min. HP 1.5% 5 ml for 2 min. PVP-I 1%, 5 ml for 2 min. Listerine, 5 ml for 2 min.	Water, 5 ml for 2 min.	RT-PCR	Rinses with Listerine or CHX can reduce the SARS-CoV-2 viral load in the oral cavity for up to two hours compared with the control.
Ferrer et al. (2021)	Spain	Randomized, multicenter, double-blind, parallel, controlled	58	Group PVP-I (n=9) Group HP (n=14) Group CPC (n=11) Group CHX (n=12) Control Group (n=12)	At the beginning of the study, 30 minutes, and it is 1 and 2 hours after rinsing.	PVP-I 2%,15 ml for 1 min. HP 1%, 15 ml for 1 min. CPC 0.07%, 15 ml for 1 min. CHX 0.12%, 15 ml for 1 min.	Distilled water,5 ml for 1 min.	RT-PCR	None of the mouthwashes significantly reduced viral load when compared with baseline. In the distilled water Control Group, a significant number of patients also experienced a decrease in viral load.
Gul et al. (2022)	Turkey	Randomized, blind, controlled	61	Group HOCl (n=20) Group PVP-I (n=21) Control Group (n=20)	At the beginning and after rinsing.	HOCl 0.02%,20 ml for 30 s. PVP-I 0.5%, 20 ml for 30 s.	Saline solution (0.9%), 20 ml for 30 s.	RT-PCR	There were no statistically significant differences between the three groups before and after rinsing.
Natto et al. (2022)	Saudi Arabia	Randomized, single-blind, parallel, controlled	60	Group CHX 1 (n=15) Group CHX 2. (n=15) Group PVP-I (n=15) Control Group (n=15)	At the beginning and immediately after rinsing.	CHX 0.12%,10 ml for 30s CHX tabs, 2mg, until they melted slowly in the mouth. PVP-I 0.5%, for 30 s.	Saline solution, 10 ml for 30 s.	RT-PCR	There were no statistically significant differences in the reduction of viral load between the test groups at both times compared with the control.
Sánchez Barrueco et al. (2022)	Spain	Randomized, double-blind, parallel, controlled	44	Group PVP-I (n=9) Group HP (n=6) Group CPC (n=10) Group CHX (n=9) Control Group (n=10)	At the beginning and after 30 minutes and 1 hour.	PVP-I 2%,15 ml for 1 min. HP 1%, 15 ml for 1 min. At the beginning and after 30 minutes and 1 hour. CHX 0.12%, 15 ml for 1 min.	Distilled water 15 ml for 1 min.	RT-PCR	None of the four types of mouthwash reduced the total viral load in saliva at any time intervals evaluated.
Sánchez Barrueco et al. (2023)	Spain	Randomized, double-blind, multicenter, parallel, controlled	23	Group CHX1 (n=8) Group CHX2 (n=5) Group CymZnCl2 (n=5) Control Group (n=5)	At the beginning, 5, 15 minutes, and 1 hour after rinsing.	CHX 0.12%, 15 ml for 1 min. CHX 0.2%, 15 ml for 1 min. CymZnCl2, 15 ml for 1 min.	Distilled water,5 ml for 1 min.	RT-qPCR	They observed a significant decrease in viral load at 15 min and 1 hour after rinsing with CHX 0.12%.
Seneviratne et al. (2021)	Singapore	Randomized, Parallel, Controlled	16	Group PVP-I (n=4) Group CHX (n=6) Group CPC (n=4) Control Group (n=2)	At the beginning, 5 minutes, 3 and 6 hours after rinsing.	PVP-I 0.5%, 5 ml for 30 s. CHX 0.2%,15 ml for 30 s. CPC 0,075%,20 ml for 30s	Sterilized water 15 ml for 30 s.	RT-PCR	CPC at 5 minutes and 6 hours and PVP-I at 6 hours had a sustained effect on reducing viral load in saliva, compared with the control.
Tarragó-Gil et al. (2020)	Spain	Randomized, multicenter, single-blind, parallel, controlled	79	Group CPC (n=39) Control Group (n=40)	At the beginning and 2 hours after rinsing.	CPC 0.07%, 15 ml for 1 min.	A substance similar in appearance to the test	RT-qPCR	No significant differences were found between the groups.

*CPC = cetylpyridinium chloride, HP = hydrogen peroxide, CHX = chlorhexidine, PVP-I = povidone-iodine

**Table 3:** Evidence profile table.

Question: [povidone-iodine mouthwashes] compared to [placebo or no intervention] for [reduction of SARS-COV2 viral load of saliva]												
Certainty assessment							Nº patients		Effect		Certainty	Importance
Nº of studies	Study desing	Risk of bias	Inconsistency	Indirectness	Imprecision	Other considerations	[povidone-iodine mouthwashes]	[placebo or no intervention]	Relative(95% CI)	Absolute(95% CI)		
Reduction of SARS-COV2 viral load in saliva (assessed with: PCR)												
3	randomised trials	very serious	not serious	not serious	not serious	none	61	44	-	SMD 4.15 SD higher (2.11 higher to 6.18 higher)	⊕⊕◯◯ Low	IMPORTANT
CI: confidence interval; SMD: standardised mean difference												
Question: [cetylpyridinium chloride mouthwashes] compared to [placebo or no intervention] for [reduction of SARS-CoV-2 viral load in saliva]												
Certainty assessment							Nº patients		Effect		Certainty	Importance
Nº of studies	Study desing	Risk of bias	Inconsistency	Indirectness	Imprecision	Other considerations	[cetylpyridinium chloride mouthwashes]	[placebo or no intervention]	Relative(95% CI)	Absolute(95% CI)		
Reduction of SARS-COV2 viral load in the saliva (follow-up: range 1 hora to 3 horas; assessed with: PCR)												
2	randomised trials	serious	not serious	not serious	serious^a^	none	90	94	-	SMD 0.07 SD lower (0.42 higher to 0.28 higher)	⊕⊕◯◯ Low^a^	IMPORTANT
CI: confidence interval; SMD: standardised mean difference												
Explanations a. It decreases one level because although the confidence interval is narrow, the value of the effect is not significant.												
Question: [chlorhexidine mouthwashes] compared to [placebo or no intervention] for [reduction of SARS-COV2 viral load in saliva]												
Certainty assessment							Nº patients		Effect		Certainty	Importance
Nº of studies	Study desing	Risk of bias	Inconsistency	Indirectness	Imprecision	Other considerations	[chlorhexidine mouthwashes]	[placebo or no intervention]	Relative(95% CI)	Absolute(95% CI)		
Reduction of SARS-COV2 viral load in saliva (assessed with: PCR)												
2	randomised trials	serious	not serious	not serious	very serious^a^	none	65	65	-	SMD 0.5 SD higher (43.32 lower to 44.32 higher)	⊕◯◯◯ Very low^a^	IMPORTANT
CI: confidence interval; SMD: standardised mean difference												
Explanations a. It decreases two levels because the confidence intervals are wide, and the value of the effect is not significant.												

## Data Availability

This manuscript is an adaptation of the corresponding thesis to obtain the academic degree of Master in Clinic Research´s Sciences at the Posgraduate School of the Universidad Privada Antenor Orrego (Trujillo, Peru). Repository link: https://repositorio.upao.edu.pe/handle/20.500.12759/50
